# Bacteriology and Surgical Technique for Nurses

**Published:** 1901-01

**Authors:** 


BACTERIOLOGY AND SURGICAL TECHNIQUE FOR NURSES. By EMILY M. A. STONEY, Superintendent of the Training School for Nurses, St. Anthony's Hospital, Rock Island, Ill., etc. Duodecimo, pp. 190. Illustrated. Philadelphia: W. B. Saunders & Co. 1900. [Price, $1.25 net.]

A somewhat extended experience in the operating room has convinced the reviewer that a subject exceedingly difficult for the nurse to grasp is the principles underlying aseptic and antiseptic surgery, and the reason for this seems to be an insufficient acquaintance with bacteriology. Doubtless others have had the same experience. To nurses who desire to perfect themselves in surgical nursing, and to surgeons who wish to place in the hands of their assistant nurses a book which will make plainer the reasons for the various routine procedures of the operating room this book is commended. M. J. F.

